# A systematic evaluation of grayscale conversion methods for mitigating color variation in deep learning-based histopathological image analysis

**DOI:** 10.1016/j.jpi.2026.100647

**Published:** 2026-02-12

**Authors:** Napat Srisermphoak, Panomwat Amornphimoltham, Risa Chaisuparat, Paniti Achararit, Todsaporn Fuangrod

**Affiliations:** a Princess Srisavangavadhana College of Medicine, Chulabhorn Royal Academy, Bangkok, Thailand; b Faculty of Dentistry, Mahidol University, Bangkok, Thailand; c Faculty of Dentistry, Chulalongkorn University, Bangkok, Thailand

**Keywords:** Digital pathology, Color variation, Grayscale, Deep learning, Image classification, Image segmentation

## Abstract

The clinical adoption of deep learning (DL) for histopathological image analysis is hindered by performance degradation caused by color variations arising from disparate staining protocols and scanning technologies. As morphological features may effectively provide the diagnostic information in hematoxylin and eosin slides, this study investigated grayscale conversion as an approach to standardize input for DL. We evaluated six grayscale algorithms against RGB across: (1) a single-center baseline, (2) a mixed multicenter training, (3) a cross-scanner generalization test, and (4) a cross-center generalization test. Furthermore, a novel attention-based grayscale conversion method (ACSRM) was investigated. It utilizes the transformer's attention mechanism to preserve critical color information through long-range pixel dependencies. In homogeneous settings, the best-performing grayscale methods achieved performance comparable to RGB (All-class F1 differences: −0.01 to 0.04 and no intersection over union differences). In mixed-center training, at least one of the grayscale algorithms outperformed RGB in every model, with 23 of 30 model combinations exhibiting statistically distinct decision behaviors (Wilcoxon signed-rank test: *p* < 0.05). Under the distribution-shift scenario, grayscale methods demonstrated better generalization: ACSRM (with Swin-Transformer-base (Swin-B)) outperformed the RGB baseline by 0.31 (0.14 and 0.45) on a specific class in cross-scanner tests, while demonstrating comparable performance on the remaining classes. Similarly, Luster with Swin-B improved F1-scores from 0.50 to 0.78 in cross-center evaluation. Statistical analysis confirmed significant differences in predictive behavior for these combinations on (3) and (4) (McNemar's Test: *p* < 0.05). Overall, ACSRM and Luster emerged as the most effective strategies for enhancing DL generalization, facilitating reliable clinical deployment.

## Highlights

• Deep learning models trained on grayscale inputs remain competitive with RGB baselines across homogeneous evaluations.
• Grayscale conversion serves as a generalization technique against scanner- and center-induced color variations.
• Top-performing grayscale models mostly exhibit statistically distinct decision functions compared to their RGB baselines.
• Luster and our novel ACSRM emerge as the most effective strategies for mitigating the color variation challenge.
• Limitations include the task-specific evaluations, the focus on RGB-grayscale comparisons, and reliance on patch sampling.

## Graphical abstract

**Figure:**
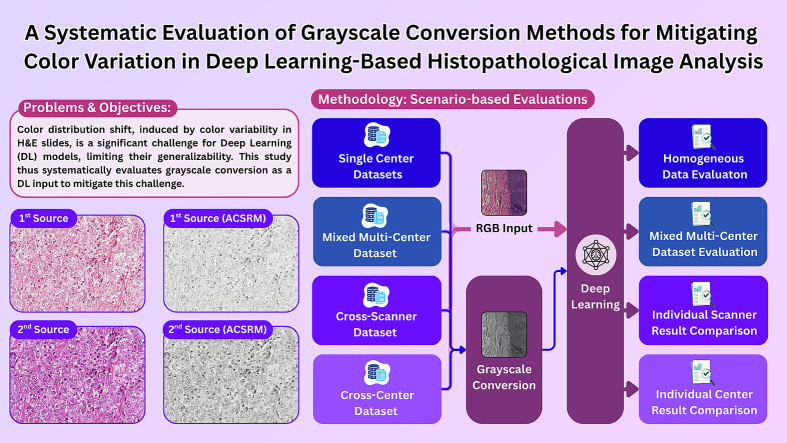

## Introduction

The adoption of whole-slide imaging (WSI), the high-resolution digital capture of tissue glass slides,^1^ has accelerated the digital transformation in pathology, including the deep learning (DL) integration. These models have demonstrated remarkable performance in assisting pathologists with labor-intensive and time-consuming analytical tasks,^2^, ^3^, ^4^, ^5^, ^6^ offering improvements in workflow efficiency and diagnostic accuracy. However, the widespread clinical adoption is hindered by a critical vulnerability: the sensitivity of DL models to data distribution shifts.^7^ This challenge results in substantial performance degradation, when these models encounter WSIs acquired under conditions different from the training data. Such distribution shifts commonly arise from variations in staining protocols, reagent concentrations, scanner manufacturers, and image acquisition environments.^1^^,^^2^^,^^8^

A primary strategy to address this challenge is stain normalization, where the color distributions of images are mapped to a standardized distribution.^9^^,^^10^ However, novel normalization techniques start to rely on complex generative models^11^ such as StainGAN, a well-known generative-based normalizer inspired by CycleGAN.^12^ Due to the inherent nature of generative DL, they risk introducing computational artifacts or may fail to generalize to unseen color distributions from new medical centers and labs, potentially creating a new set of downstream challenges. Another approach, also the focus of this study, is to remove color information entirely through grayscale conversion. This strategy is promising because hematoxylin and eosin (H&E) staining primarily reveals diagnostic information through morphological and structural features with the limited color palette—typically blue/purple for nuclei and pink for tissue structures.^8^ We thus hypothesize that grayscale conversion can serve as a robust standardization method at the cost of sacrificing color-specific diagnostic information.^8^^,^^10^

Grayscale conversion is standard in image processing, valued for its ability to reduce computational complexity and simplify subsequent analysis. It has primarily served as a foundational preprocessing step for two applications: tissue localization and traditional feature extraction.^3^^,^^4^^,^^13^, ^14^, ^15^, ^16^, ^17^, ^18^ Tissue localization is a process for isolating tissue regions from the extensive background in a WSI.^19^ For instance,^17^ tested multiple grayscale/dimensional-reduction methods (i.e., luminosity, saturation channel extraction, color distance, and local entropy) with Otsu's and fixed thresholding on a tissue segmentation task. These combinations mostly obtained median Intersection over Union (IoU) scores above 0.8. Additionally, Bándi et al.^18^ applied luminance-based grayscale images with foreground extraction from structure information (FESI) to perform tissue segmentation. They compared their method with DL models trained on RGB images and found that whereas the DL models scored higher with a mean Jaccard Index between 0.93 (±0.06) and 0.94 (±0.06), the FESI method still performed competitively with a Jaccard Index of 0.87 (±0.15).

Traditional feature extraction often relies on grayscale images to derive numerical descriptors that capture textural and structural patterns. These features are generally applied to traditional machine learning (ML). For instance, Xu et al.^14^ utilized red-channel-based textural features, derived from variants of local binary pattern (LBP) and other texture descriptors, with support vector machines (SVM). They achieved promising performance for prostate cancer grading at a minimum classification accuracy of 0.72.^3^ applied grayscale-based structural features (e.g., clump thickness and cell size uniformity) with ML models for breast cancer classification, and all obtained accuracy above 0.9. Belsare et al.^16^ used a set of first-order intensity, gray level *co*-occurrence matrix (GLCM), graph run length matrix (GRLM), and the morphological Euler number in linear discriminant analysis for breast cancer classification. In addition, Nateghi et al.^15^ integrated structural, textural, and graph-based features from gland subregions into an SVM for gland segmentation, achieving an F1-score of 0.68. Lastly, He et al.^13^ fused GLCM and LBP with convolutional neural network (CNN)-based color features from pretrained AlexNet and GoogLeNet, and fit to SVM for breast cancer classification. It achieved accuracies approaching 0.99.

Despite its success in traditional ML, the direct utilization of grayscale images as input to DL models has remained underexplored. Previous studies^5^^,^^6^ applied grayscale images to DL using the Kimia Path24 dataset. Kieffer et al.^6^ performed transfer learning on the Inception-v3, achieving a patch classification accuracy of 0.75. Similarly, another study^5^ conducted transfer learning on different pretrained CNNs, i.e., ResNet50 and DenseNet-161, demonstrating the patch accuracy of more than 0.96. This score range is also considered the same level of performance as color-input models. Lastly, Rezaei et al.^4^ applied red-channel images, grayscale-based features derived from invariant LBP, and hematoxylin component features to a DL-based segmentation architecture for gland segmentation. They illustrated object-level Dice scores of 0.82 and 0.87 across different testing datasets. In the specific context of color variation, Tellez et al.^20^ remains the only study to explicitly observe the impact of grayscale inputs on DL generalization. They evaluated various stain color augmentation and normalization techniques (e.g., grayscale, network-based mapping, generative-based approach, and Macenko's method^21^) on CNN classification. Although the authors concluded that normalization is generally complementary to augmentation, the choice of normalization method remains critical, as it establishes the baseline input for the augmentation process. To isolate the impact of normalization, we further analyzed their reported performance under their basic augmentation setting (rotation and flipping) and found that grayscale normalization achieved performance second only to their proposed network-based method. This suggests that grayscale images hold promise as a robust foundation for DL, yet the specific impact of different grayscale conversion algorithms remains unstudied.

To our knowledge, no work has been found to systematically and dedicatedly investigate whether training DL models on specific grayscale images can improve their robustness and generalization when encountered with color variations. This study thus addresses this gap by conducting a systematic evaluation of grayscale conversion methods, as individual methods preserve different aspects of color images,^22^, ^23^, ^24^ against an RGB baseline for mitigating color variation in DL-based histopathological image analysis. To achieve systematic exploration, we designed four experimental scenarios simulating clinical challenges: (1) baseline performance on single-center data, (2) training generalization on a mixed multicenter, (3) generalization on images from a different scanner, and (4) generalization on images from a different center. Furthermore, we assess our proposed attention-based grayscale conversion method (ACSRM) against other techniques.^25^ We designed it to capture spatial relationships among RGB channels by leveraging the transformer's attention mechanism to maximize color information preservation. Through this comprehensive investigation, we aim to provide definitive insights into the utility of grayscale conversion as a practical and effective strategy for developing more robust and deployable DL solutions for digital pathology.

## Methodology

### Datasets

#### Atypia14^26^

A patch-based dataset for nuclear atypia grade classification in breast cancer H&E images. Patch images are categorized into three classes, including low-, moderate-, and high-grade atypia as classes 0, 1, and 2, respectively. This dataset's defining feature is that each patch is available in two versions, which were acquired from two different scanners (i.e., an Aperio Scanscope XT and a Hamamatsu Nanozoomer 2.0-HT). This characteristic offers a controlled environment to assess model robustness to distribution shifting from scanner variability.

#### PANDA^27^

A WSI dataset for prostate cancer segmentation. It was sourced from two centers, i.e., the Karolinska Institute and Radboud University Medical Center. Due to annotation discrepancies between the sources, we repurposed this dataset for a binary classification task (cancerous and non-cancerous tissue), allowing for the evaluation of model performance under acquisition environment variations.

#### CAMELYON16^28^

A breast cancer metastasis segmentation dataset was sourced from Radboud University Medical Center and University Medical Center Utrecht, consisting of 270 training and 130 testing WSIs. This multicenter nature allows the mixed multicenter training data evaluation.

#### Oral epithelium segmentation (OES)^19^

A segmentation dataset was developed for oral epithelium segmentation, consisting of 7 H&E WSIs collected under a single environment. Ethical approval was granted by the Chulabhorn Royal Academy's research ethics committee (EC 040/2567).

### Data preparation pipeline

#### Atypia14

The images were first resized to match the dataset's minimum dimensions and subsequently downscaled by a factor of four to a resolution of 384 × 344. For training, we utilized images exclusively from the Aperio scanner, reserving five patches per class for validation. To address significant class imbalance in the training partition (Class 1: *n* = 222 vs. Class 2: *n* = 52 and Class 0: *n* = 23), we applied data augmentation (horizontal and vertical flipping) to balance the minority classes. Similarly, a disparity also exists in the predefined testing partitions, which comprise 60 samples for Class 1, 38 for Class 0, and 22 for Class 2.

#### PANDA

*We* first merge background and benign into the non-cancer class, whereas cancer-related classes are consolidated into one class to avoid the annotation inconsistencies. We then selected a subset of WSIs for our experiment by randomly selecting 50 WSIs from the Karolinska Institute source on each ISUP grade (0–5) for training. The test set was composed of five randomly selected WSIs from each of the two sources. We then applied a conditional random patch extraction to generate a dataset of non-overlapping patches. The target for each WSI was to extract ten 224 × 224 patches for each class present, subject to several criteria: (1) a background area <50%, (2) a cancer area <30% and a benign area ≥50% for non-cancerous patches, and (3) a cancer area ≥30% for cancerous patches. If the target of 10 patches could not be met, the target number was iteratively reduced. If it reached zero, overlapping patches were permitted. Finally, 10% of the training images were randomly selected for validation.

#### CAMELYON16

To ensure high-quality ground truth and prevent label noise, we first exclude unexhaustively annotated WSIs, followed by merging background and normal into a non-cancer class. We then applied the previous patching strategy with minor adjustments to create a segmentation dataset: (1) a background area <70%, and (2) a cancer area <20% and a normal tissue ≥30% for non-cancerous patches. This extraction produced 10 non-cancerous and 30 cancerous patches per WSI from the training and predefined testing sets. Additionally, 10% of the training patches were reserved for validation.

#### OES

Following the protocol from,^19^ Binary Otsu's thresholding was first conducted on resized WSIs to locate individual tissues, followed by a postprocessing sequence. Consequently, a total of 49 whole-tissue images were extracted, divided into 40 training, 5 validation, and 4 testing images. To ensure robust evaluation results, we created three variations of this data split through random resampling. Lastly, individual images were then non-overlappingly patched to create a patch dataset, where every patch shared a resolution of 224 × 224.

### Grayscale conversion techniques

#### Intensity

This approach^23^ converts color images to grayscale by averaging the values across the RGB channels

$$\frac{R+G+B}{3}.$$

#### Luster

This method^23^ represents grayscale images using the L channel of the HLS color format, which consists of hue (H), lightness (L), and saturation (S) channels:

$$\frac{1}{2}\left(\max\left(R,G,B\right)+\min\left(R,G,B\right)\right).$$

#### Luminosity

Because human visual perception is particularly sensitive to green compared to other colors,^29^ the luminosity approach leverages this attribute by using a linear equation that assigns the highest weight to the green channel in the RGB format:

0.21R+0.72G+0.07B.

#### Luminance

Open-source computer vision libraries^23^ commonly deliver this grayscale conversion approach. In this study, both CV2 and PyTorch libraries are chosen for the experiments based on the assumption that they are likely to be used when no specific grayscale conversion method is prioritized:

0.3R+0.59G+0.11B.

#### Attention-based grayscale conversion method (ACSRM)^25^

Proposed in our previous study, it exploits the attention mechanism to capture the long-range dependencies to derive a relationship between individual pixels across the RGB color channels.

$$\frac{1}{3}∙1^{T}∙\left(\mathrm{Softmax}\left(\frac{FF^{T}-\mu_{\mathrm{row}}\left(FF^{T}\right)}{\sigma_{\mathrm{row}}\left(FF^{T}\right)}\right)∙F\right)$$

Where F is a matrix of color-channel flattened vectors derived from an input image, $\mu_{\mathrm{row}}\left(FF^{T}\right)$ and $\sigma_{\mathrm{row}}\left(FF^{T}\right)$ represent color-channel-relationship-wise means and standard deviations to perform standardization.

### Experiment schemes

**Fig. 1:**
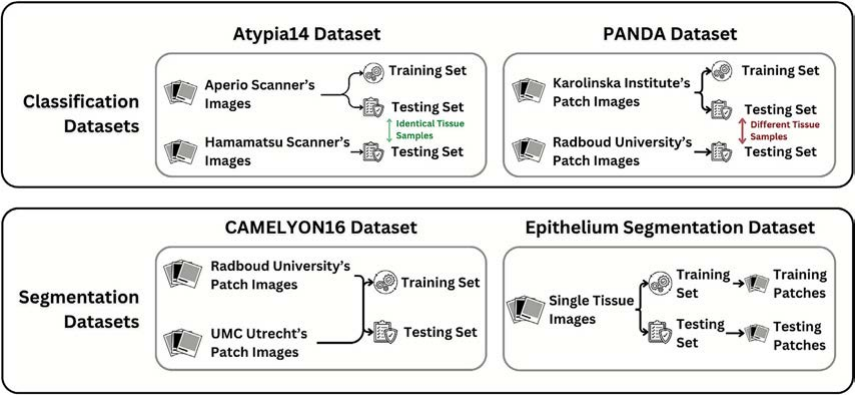
Characteristics of the training and testing sets in the Atypia14, PANDA, CAMELYON16, and OES datasets.

To systematically assess the impact of grayscale conversion as DL inputs against the RGB baseline, we designed four experiments to simulate clinical challenges. (see Fig. 1).

#### Baseline performance on single-center data

An experiment established a performance baseline on homogeneous datasets, aiming to determine whether models trained on grayscale inputs could perform comparably to their RGB counterparts when no significant domain shift was present. We thus used Aperio's images on Atypia14, Karolinska's images on PANDA, and the entire OES dataset to explore this scenario.

#### Training generalization on a mixed multicenter

An experiment investigated DL models when trained and tested on images sourced from multiple centers to evaluate learning abilities and robustness of DL candidates. We thus used the CAMELYON16 for this scenario.

#### Cross-scanner generalization

An experiment explored cross-scanner generalization of DL candidates when encountering shifted distribution inputs, caused by different scanner utilization. We utilized the Atypia14 dataset by using Aperio's images for training and Hamamatsu's for testing.

#### Cross-center generalization

An experiment examined cross-center generalization, evaluating how well models perform when encountering images from an entirely new medical center. The PANDA dataset was thus utilized by training on Karolinska's images and testing on Radboud's images in this scenario. However, as the dataset's images from each source are completely independent, we thus set the top-performing RGB models as a baseline to deliver the grayscale performances.

### Deep learning architectures

To demonstrate the impact of color variation, we selected DL architectures from both CNN and transformer families. These models were chosen as they employ distinct mechanisms for feature extraction and learning. CNN extracts local features via a sliding window, whereas Vision Transformer (ViT) analyzes global context via the relationships among image patches.^30^^,^^31^

Consequently, we selected VGG19 and ResNet50 variants as representative CNN architectures due to their widespread adoption and established performance benchmarks. Whereas standard ViT models typically require large volumes of data to converge due to a lack of inductive bias, we selected the Swin Transformer to mitigate this limitation.^32^ It incorporates hierarchical inductive biases, thereby alleviating the data-hungry behavior while retaining global context capabilities. Accordingly, the classification architectures employed in this study included ResNet50, VGG19, and the Swin-Transformer-base (Swin-B). These models were implemented using the PyTorch library. For the segmentation tasks, UNet, ResNet50-UNet, and VGG19-UNet were selected as CNN-based standard and selected variant candidates (sourced from^33^^,^^34^), whereas SwinUNet and MISSFormer were chosen to represent transformer-based architectures (sourced from^35^^,^^36^).

### Training parameter settings and hardware specification

All experiments were conducted under a standardized training protocol to ensure a fair comparison. Prior to training and inference, images from all domains (i.e., color and grayscale variants) underwent channel-wise standardization.

In training, we consistently utilized the Adaptive Moment Estimation (Adam) optimizer with a batch size of 24. Data augmentation of horizontal and vertical flips was also applied. We restricted the augmentation to these geometric transformations because we need to isolate the intrinsic sensitivity of the models to natural color distribution shifts, ensuring that synthetic color perturbations did not mask the performance differences between grayscale and color modalities. Regarding loss functions, cross-entropy (CE) loss was employed for the multiclass task (i.e., Atypia14), whereas binary cross-entropy (BCE) loss was utilized for the binary classification and segmentation tasks (i.e., PANDA, CAMELYON16, and OES). The training epochs were set to 100 as a standard; however, this was extended to 300 epochs for the Atypia14 dataset to accommodate the convergence difficulties associated with multiclass classification. Learning rates were adjusted according to task complexity. A lower learning rate of 1e-5 was applied to the classification datasets to ensure training stability, given the class imbalance on Atypia14, and to keep consistency on PANDA, whereas the segmentation tasks utilized a learning rate of 1e-3. (see Table 1).

**Table 1:** Training parameter settings for individual classification and segmentation datasets.

Datasets	Loss function	Optimizers	Epochs	Learning rate	Batch size
Atypia14	CE	ADAM	300	1e-5	24
PANDA	BCE	ADAM	100	1e-5	24
CAMELYON16	BCE	ADAM	100	1e-3	24
OES	BCE	ADAM	100	1e-3	24

In terms of hardware specifications, all experiments were conducted on a workstation configured with Microsoft Windows 11 Pro and Python version 3.12.3. The hardware is centered around a CPU of 13th Gen Intel Core i9-13900K (3.00 GHz). For graphical computations and neural network acceleration, the system employs an NVIDIA RTX 4090 GPU.

### Classification's evaluation metrics

Due to the significant class imbalance in the Atypia14 dataset, accuracy can be biased toward the majority class. Consequently, the classification tasks were evaluated using precision, recall, and F1-score. Precision measures the accuracy of positive predictions, specifically how effectively each architecture identifies positive-class images. Recall evaluates the ability to retrieve all actual positive-class images. The F1-score serves as the harmonic mean of precision and recall. The equations of the three remaining metrics are as follows:

$$\mathrm{Precision}=\frac{\mathrm{TP}}{\mathrm{TP}+\mathrm{FP}}$$

$$\mathrm{Recall}=\frac{\mathrm{TP}}{\mathrm{TP}+\mathrm{FN}}$$

$$F1-\mathrm{score}=2∙\frac{\mathrm{Precision}∙\mathrm{Recall}}{\mathrm{Precision}+\mathrm{Recall}}$$

Where true positive (TP) refers to correctly identifying positive-class images, false positive (FP) describes incorrectly classifying negative-class images as positive, and false negative (FN) occurs when positive-class images are incorrectly categorized as negative.

Furthermore, to provide an additional evaluation of the model's discriminative capability, we employed the area under the receiver operating characteristic curve (AUC). The ROC curve plots the TP rate against the FP rate, where an AUC of 1.0 indicates a perfect classifier, whereas an AUC of 0.5 represents random guessing.

To statistically validate performance differences between RGB and grayscale-variant models across the color-variation scenarios (i.e., Atypia14 and PANDA), we utilized the two-tailed McNemar's test to assess the significance of discordant predictions with a confidence level of 95%. This test was selected because it is specifically designed for paired nominal data (i.e., binary correct/incorrect outcomes), allowing us to robustly compare classifiers on a single test set.

### Segmentation's evaluation metrics

Each segmentation architecture is evaluated based on IoU, also known as the Jaccard Index, and the dice similarity coefficient (DSC). IoU quantifies the overlap between predicted outputs and ground-truth images, whereas DSC quantifies the similarity between the two masks.

$$\mathrm{IoU}=\frac{\left|A\cap B\right|}{\left|A\cup B\right|}$$

$$\mathrm{DSC}=\frac{2∙\left|A\cap B\right|}{\left|A\right|+\left|B\right|}$$

Where A is a prediction output from each architecture, and B is a ground-truth mask.

To statistically compare segmentation performance between RGB and grayscale-variant models in the color-variation scenario (i.e., CAMELYON16), we employed the Wilcoxon signed-rank test with a 95% confidence level. This test was selected for its suitability for paired continuous data without normality assumptions, allowing us to robustly assess performance differences in IoU scores while accounting for the non-normal distribution often caused by various segmentation difficulty across samples.

## Results

### Baseline performance on single-center data

#### Atypia14

As shown in Table 2 (Aperio's results), performance on the severe atypia (Class 2) was challenging for all combinations. The best-performing RGB model was VGG19, achieving 0.46, 0.71, and 0.44 F1-scores on mild, moderate, and severe atypia, respectively. Three grayscale combinations mostly reached comparable performance on the mild and moderate classes, while maintaining acceptable performance on the remaining class: Luster with ResNet50 and ACSRM with ResNet50 and Swin-B. For the most challenging severe atypia class, the ACSRM with Swin-B model obtained the highest F1-score among all grayscale methods at 0.32.

#### PANDA

The evaluation result is illustrated in Table 3 (Karolinska's results). The top-performing RGB models were VGG19 and Swin-B, both achieving a shared F1-score of 0.69. This score was slightly surpassed by two grayscale combinations, including ACSRM with VGG19 and Luster with Swin-B at F1-score of 0.71 and 0.7, respectively. These combinations also demonstrated higher recall scores compared to the VGG19 baseline.

#### OES

Table 4 illustrates the evaluation results of the OES task. The best-performing RGB was UNet, achieving an IoU of 0.82 and a DSC of 0.87, considered the highest scores across all combinations. However, these baseline scores were also demonstrated by Luster with UNet and ACSRM with UNet.

#### Overall

These evaluations predominantly demonstrated that grayscale conversion techniques performed comparably to, and in some cases slightly surpassed, the performance of the individual best-performing RGB baselines. This key finding confirms that the morphological and structural features extracted through DL layers are sufficient for performing the histopathological tasks accurately under homogeneous conditions.

### Training generalization on a mixed multi-center dataset

This scenario-based experiment was explored through the CAMELYON16 dataset and reported in Table 5. Overall, the best-performing RGB model was ResNet50-UNet, demonstrating 0.7 IoU and 0.76 DSC. However, the highest scores were obtained by Luminance (Torch) with UNet (0.72 IoU and 0.80 DSC), followed closely by Luster with VGG19-UNet and ACSRM with UNet. Moreover, there are several combinations illustrating competitive performance to the RGB baseline.

**Fig. 2:**
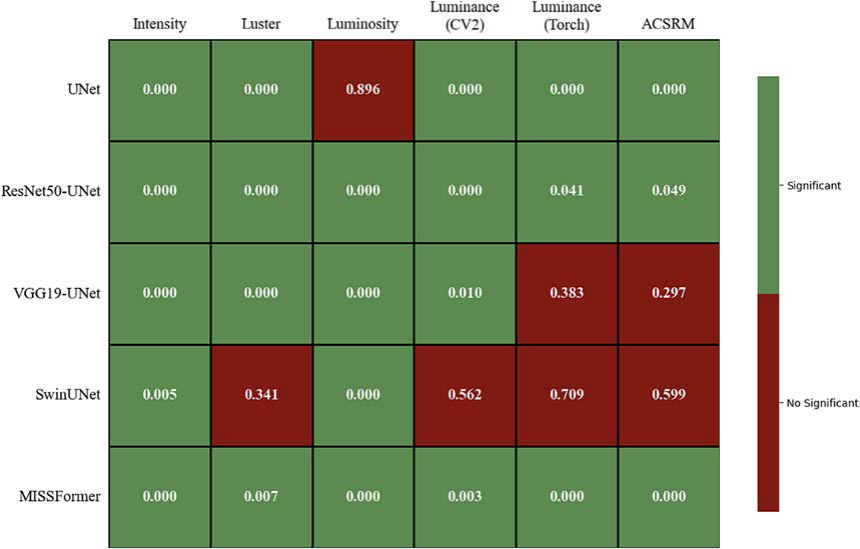
Statistical significance heatmap of segmentation performance on CAMELYON16. Pairwise comparison of grayscale-variant models against their RGB counterparts using the Wilcoxon signed-rank test. Green indicates significant differences (*p* < 0.05), whereas red indicates no significant difference. (For interpretation of the references to color in this figure legend, the reader is referred to the web version of this article.)

To statistically validate these observations, the Wilcoxon signed-rank test was conducted on the IoU scores to compare performance differences between RGB and the grayscale formats across all architectures. As illustrated in Fig. 2, 23 out of 30 grayscale combinations demonstrated a statistically significant difference (*p* < 0.05) against their RGB counterparts, indicating distinct prediction functions compared to their baselines. In terms of absolute score, at least one of these statistically significant combinations provided performance comparable to or significantly better than the RGB baseline in every evaluated DL architecture.

According to these results, sacrificing color information and forcing the models to rely on morphological and structural features not only leads to different DL decision functions but also arguably provides better capability in recognizing essential patterns from the H&E images.

### Cross-scanner generalization

This experiment assessed the generalization abilities of DL combinations when encountering distribution shifts caused by utilizing a different scanner during image collection. To simulate a real-world deployment situation, the top-performing combinations (i.e., champions) from the Aperio evaluation were selected to evaluate on the cross-scanner test, i.e., RGB-VGG19 (RGB baseline), Luster-ResNet50, and ACSRM with ResNet50 and Swin-B.

As shown in Table 2, the RGB baseline experienced a sharp performance decline in both moderate (Class 1) and severe (Class 2) atypia for 0.32 (0.71–0.39; −45%) and 0.3 (0.44–0.14; −68%), respectively. A similar trend was also illustrated in the grayscale candidates on low and moderate atypia, but with a significantly different consequence in the severe atypia, especially on ACSRM combinations, where F1-scores increased to 0.37 and 0.45. On the moderate atypia, ACSRM combinations illustrated an equivalent performance level to the RGB baseline at approximately 0.4, whereas the Luster combination struggled at 0.24 F1-score. However, the Luster candidate led the highest F1-score on the low atypia (i.e., Class 0) with 0.48 F1-score, followed by the RGB baseline and ACSRM candidates.

**Table 2:** F1-scores and AUC scores for DL classification models on the Atypia14 dataset.

Scanners		Aperio					Hamamatsu				
Architectures	Format	Classes			Avg.	AUC	Classes			Avg.	AUC
		0	1	2			0	1	2		
ResNet50	RGB	0.43	0.69	0.08	0.40	0.60	0.37	0.53	0.00	0.30	0.55
	Intensity	0.46	0.69	0.08	0.41	0.64	0.38	0.47	0.22	0.36	0.57
	Luster	**0.47**	**0.70**	**0.23**	**0.47**	**0.62**	**0.48**	**0.24**	**0.24**	**0.32**	**0.57**
	Luminosity	0.45	0.64	0.00	0.36	0.63	0.54	0.38	0.16	0.36	0.61
	Luminance (CV2)	0.42	0.70	0.15	0.42	0.64	0.42	0.39	0.21	0.34	0.58
	Luminance (Torch)	0.41	0.64	0.13	0.39	0.66	0.48	0.24	0.35	0.36	0.58
	ACSRM	**0.41**	**0.70**	**0.21**	**0.44**	**0.68**	**0.27**	**0.40**	**0.37**	**0.34**	**0.59**
VGG19	RGB	**0.46**	**0.71**	**0.44**	**0.53**	**0.67**	**0.37**	**0.39**	**0.14**	**0.30**	**0.55**
	Intensity	0.37	0.64	0.00	0.34	0.65	0.38	0.46	0.37	0.40	0.58
	Luster	0.43	0.67	0.16	0.42	0.64	0.40	0.22	0.40	0.34	0.55
	Luminosity	0.35	0.65	0.00	0.33	0.63	0.47	0.29	0.18	0.31	0.57
	Luminance (CV2)	0.38	0.65	0.00	0.34	0.64	0.39	0.36	0.35	0.36	0.56
	Luminance (Torch)	0.39	0.66	0.00	0.35	0.63	0.42	0.35	0.14	0.31	0.56
	ACSRM	0.45	0.70	0.08	0.41	0.61	0.19	0.37	0.45	0.34	0.54
Swin-B	RGB	0.42	0.71	0.08	0.40	0.62	0.39	0.11	0.00	0.17	0.41
	Intensity	0.13	0.70	0.06	0.30	0.59	0.08	0.50	0.15	0.24	0.50
	Luster	0.38	0.70	0.08	0.39	0.61	0.32	0.38	0.24	0.31	0.54
	Luminosity	0.13	0.69	0.06	0.29	0.57	0.12	0.37	0.13	0.21	0.45
	Luminance (CV2)	0.13	0.69	0.06	0.29	0.58	0.03	0.40	0.14	0.19	0.46
	Luminance (Torch)	0.13	0.69	0.06	0.29	0.58	0.03	0.40	0.14	0.19	0.46
	ACSRM	**0.42**	**0.71**	**0.32**	**0.49**	**0.65**	**0.32**	**0.39**	**0.45**	**0.39**	**0.62**

**Bold numbers** represent grayscale combinations illustrating competitive performance against an RGB baseline on the homogenous testing set (i.e., Aperio's testing images).

In addition, McNemar's statistical testing was conducted between the RGB baseline and the grayscale champions to evaluate the significance of discordant predictions (Table 6). Luster with ResNet50 exhibited statistically significant differences in classes 0 and 1 (*p < 0.05*). However, it only demonstrated superiority in Class 0, while significantly underperforming the RGB baseline in Class 1. Similarly, ACSRM with ResNet50 showed significant differences in classes 0 and 2 (*p < 0.05*), but provided a performance advantage only in Class 2 with a notable degradation in Class 0. In contrast, ACSRM with Swin-B only achieved statistically significant superiority in Class 2, while maintaining performance parity with the RGB baseline in classes 0 and 1. Notably, this performance was considered the top-tier performance among the champions.

Accounting for this evidence, ACSRM with Swin-B proved to be the best generalized model, achieving balanced F1-scores of 0.32 (low), 0.39 (moderate), and 0.45 (severe), and the highest overall AUC of 0.62. Its unique ability to significantly recover performance in the severe class without compromising performance in others confirms its utility as a robust safety mechanism for this scenario.

### Cross-center generalization

This experiment explored the best-performing DL combinations from Karolinska's results in generalizability on unseen images collected from a different center (Radboud's images). The candidates (i.e., champions) included RGB with VGG19 and Swin-B, Luster with Swin-B, and ACSRM with VGG19. The results were reported in Table 3.

**Table 3:** Performance results for DL classification models on the PANDA dataset.

Data sources		Karolinska Institute				Radboud University			
Architectures	Format	Precision	Recall	F1	AUC	Precision	Recall	F1	AUC
ResNet50	RGB	0.66	0.65	0.66	0.73	0.53	0.23	0.32	0.65
	Intensity	0.66	0.47	0.55	0.68	0.54	0.71	0.62	0.64
	Luster	0.63	0.62	0.62	0.71	0.57	0.67	0.62	0.67
	Luminosity	0.67	0.47	0.55	0.67	0.54	0.60	0.57	0.59
	Luminance (CV2)	0.64	0.64	0.64	0.70	0.52	0.72	0.60	0.58
	Luminance (Torch)	0.63	0.61	0.62	0.68	0.54	0.77	0.63	0.61
	ACSRM	0.64	0.70	0.67	0.72	0.63	0.56	0.59	0.70
VGG19	RGB	**0.73**	**0.65**	**0.69**	**0.78**	**0.45**	**0.06**	**0.11**	**0.38**
	Intensity	0.67	0.64	0.65	0.73	0.76	0.64	0.70	0.81
	Luster	0.74	0.62	0.67	0.78	0.74	0.58	0.65	0.77
	Luminosity	0.69	0.58	0.63	0.74	0.72	0.80	0.76	0.83
	Luminance (CV2)	0.67	0.64	0.65	0.74	0.73	0.75	0.74	0.84
	Luminance (Torch)	0.73	0.50	0.60	0.75	0.82	0.67	0.74	0.83
	ACSRM	**0.71**	**0.71**	**0.71**	**0.77**	**0.71**	**0.41**	**0.52**	**0.72**
Swin-B	RGB	**0.68**	**0.71**	**0.69**	**0.77**	**0.62**	**0.42**	**0.50**	**0.61**
	Intensity	0.70	0.60	0.64	0.73	0.70	0.88	0.78	0.87
	Luster	**0.71**	**0.69**	**0.70**	**0.78**	**0.69**	**0.91**	**0.78**	**0.87**
	Luminosity	0.67	0.62	0.65	0.73	0.71	0.85	0.77	0.87
	Luminance (CV2)	0.66	0.65	0.66	0.74	0.69	0.88	0.78	0.88
	Luminance (Torch)	0.66	0.65	0.66	0.74	0.69	0.89	0.78	0.88
	ACSRM	0.68	0.58	0.63	0.74	0.66	0.86	0.75	0.83

**Bold numbers** represent grayscale combinations demonstrating competitive performance against an RGB baseline on the homogenous testing set (i.e., Karolinska Institute's testing images).

**Table 4:** Performance results for DL segmentation models on the OES dataset.

	RGB		Intensity		Luster		Luminosity		Luminance (CV2)		Luminance (Torch)		ACSRM	
Architectures	IoU	DSC	IoU	DSC	IoU	DSC	IoU	DSC	IoU	DSC	IoU	DSC	IoU	DSC
UNet	**0.82**	**0.87**	**0.80**	**0.86**	**0.82**	**0.87**	0.79	0.86	**0.80**	**0.86**	**0.80**	**0.86**	**0.82**	**0.87**
ResNet50-UNet	0.77	0.82	0.74	0.80	0.74	0.80	0.72	0.78	0.74	0.79	0.77	0.83	0.76	0.82
VGG19-UNet	**0.82**	**0.86**	**0.80**	**0.86**	0.78	0.84	0.79	0.86	**0.80**	**0.86**	0.76	0.83	0.78	0.84
SwinUNet	0.77	0.83	0.68	0.76	0.71	0.79	0.68	0.77	0.68	0.76	0.67	0.75	0.70	0.78
MISSFormer	0.70	0.75	0.68	0.74	0.74	0.79	0.60	0.66	0.63	0.69	0.61	0.66	0.69	0.74

**Bold numbers** represent grayscale combinations demonstrating competitive performance against an RGB baseline on the testing set.

**Table 5:** A table of DL segmentation architectures' performances on the CAMELYON16 dataset.

	RGB		Intensity		Luster		Luminosity		Luminance (CV2)		Luminance (Torch)		ACSRM	
Architectures	IoU	DSC	IoU	DSC	IoU	DSC	IoU	DSC	IoU	DSC	IoU	DSC	IoU	DSC
UNet	0.64	0.72	**0.71**	**0.78**	**0.71**	**0.78**	0.66	0.74	**0.71**	**0.79**	**0.72**	**0.80**	**0.72**	**0.79**
ResNet50-UNet	**0.70**	**0.76**	0.66	0.73	0.63	0.70	0.67	0.74	0.67	0.74	**0.70**	**0.77**	**0.70**	**0.76**
VGG19-UNet	0.68	0.74	**0.71**	**0.78**	**0.72**	**0.79**	0.63	0.70	0.67	0.74	0.68	0.74	0.69	0.76
SwinUNet	0.60	0.70	0.64	0.75	0.63	0.74	0.67	0.76	0.62	0.73	0.62	0.73	0.63	0.74
MISSFormer	0.57	0.62	0.67	0.74	0.58	0.64	0.64	0.70	0.54	0.59	0.61	0.68	0.62	0.68

**Bold numbers** represent grayscale combinations demonstrating competitive performance against an RGB baseline on the testing set.

**Table 6:** Statistical evaluation of classification generalization on the unfamiliar Atypia14 dataset (i.e., Hamamatsu Scanner) using McNemar's test comparing the RGB baseline against grayscale champions.

Architecture	Format	Classes	Both correct	Only RGB correct	Only grayscale correct	Both incorrect	*p*-value
ResNet50	Luster	**0**	13*	1*	11*	13*	<0.05*
		**1**	**8**	**16**	**3**	**33**	**<0.05**
		**2**	1*	2*	5*	14*	>0.05*
	ACSRM	**0**	**7**	**7**	**0**	**24**	**<0.05**
		**1**	8	16	11	25	>0.05
		**2**	3*	0*	14*	5*	<0.05*
Swin-B	ACSRM	**0**	11	3	0	24	>0.05
		**1**	17	7	4	32	>0.05
		**2**	3*	0*	11*	8*	<0.05*

**Bold numbers** represent grayscale combinations demonstrating a statistically significant difference (*p* < 0.05) against the RGB baseline. An asterisk (*) indicates cases where the grayscale format performed better than the RGB baseline in absolute scores.

**Table 7:** Statistical evaluation of classification generalization on the unfamiliar PANDA dataset (i.e., Radboud University) using McNemar's test comparing the RGB baseline against grayscale champions.

Architecture	Format	Both correct	Only RGB correct	Only grayscale correct	Both incorrect	*p*-value
VGG19	ACSRM	247	79	97	111	> 0.05
Swin-B	Luster	262*	64*	147*	61*	< 0.05*

**Bold numbers** represent grayscale combinations demonstrating a statistically significant difference (*p* < 0.05) against the RGB baseline. An asterisk (*) indicates cases where the grayscale format performed better than the RGB baseline in absolute scores.

Given that the testing sources were completely independent, we established RGB with Swin-B as the baseline for performance comparison, based on its heterogeneous testing scores of 0.62 precision, 0.42 recall, and 0.5 F1-score. On the grayscale combinations, these scores were also reached by the ACSRM candidate with significant improvement in precision at 0.71 and AUC at 0.72, whereas Luster with Swin-B delivered the most impressive results with 0.69 precision, 0.91 recall, 0.78 F1-score, and 0.87 AUC.

In addition, McNemar's statistical testing was conducted to assess the significance of discordant predictions between the RGB baseline and the grayscale champions (Table 7). The analysis revealed that a statistically significant difference in decision functions (*p* < 0.05) emerged only with the Luster (Swin-B) combination. In contrast, although the ACSRM candidate demonstrated improved precision and AUC on the unfamiliar distribution, it did not reach statistical significance in terms of overall prediction discordance compared to the baseline.

According to the substantial improvement in F1-score (from 0.50 to 0.78), AUC (from 0.61 to 0.87) and the statistical confirmation, Luster with Swin-B was identified as the most generalized model overcoming the baseline for the cross-center scenario.

## Discussion

This study conducted a systematic exploration of the generalizability of grayscale converted images in mitigating color variations in DL-based histopathological image analysis. According to the first and second scenarios, we found that morphological and structural features are sufficient for DL to recognize crucial patterns within the H&E images. The grayscale combinations consistently delivered performance competitive with, and sometimes superior to, individual RGB baselines, regardless of whether the training data originated from single or multiple sources. Additionally, there is evidence that grayscale combinations represented enhanced generalization in both cross-scanner and cross-center scenarios (i.e., third and fourth scenarios), where all these champions illustrated statistically distinct prediction functions compared to their RGB baselines. These findings suggest that whereas the color provided by H&E staining is an indispensable visual aid for pathologists, the underlying textural information preserved by a grayscale conversion is often sufficient for DL models to achieve high performance and generalize against color variation.

### Relationship between grayscale conversion and color variation

Although grayscale images produced by various conversions may appear similar to the human eye, computer vision algorithms interact with them differently.^22^, ^23^, ^24^ We thus showed the differences between the outputs of each grayscale and also visually illustrated the impact of color variation. In addition, we provided a possible rationale for why grayscale conversions are particularly effective inputs for DL models in H&E images.

**Fig. 3:**
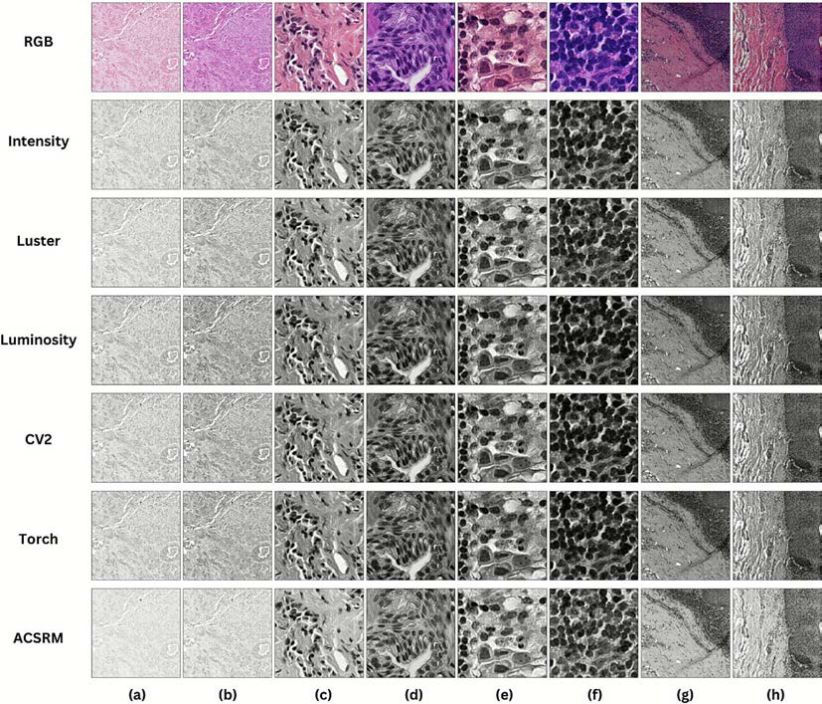
Visual comparison of RGB images and corresponding grayscale outputs from different conversion methods across datasets, where (a–b) images from the Atypia14 dataset, acquired using Aperio and Hamamatsu scanners; (c–d) images from the PANDA dataset, obtained from Radboud University Medical Center and the Karolinska Institute; (e–f) images from the CAMELYON16 dataset; and (g–h) images from the OES dataset.

Examples of grayscale images converted by our conversion candidates were visualized in Fig. 3. The influence of color variation caused by different scanners (Fig. 3a–b) and centers (Fig. 3c–f) was clearly shown on the RGB row through significant differences in color intensity and hue. This difference was visually reduced via grayscale applications. Based on grayscale output characteristics, the grayscale candidates can be categorized into three groups. The first group included only ACSRM. It produced brighter outputs while maintaining visibility in darker regions, aiding in the separation between nuclei and tissue structures. Intensity and Luster were identified as the second group. They generated grayscale representations with balanced texture preservation. Lastly, luminosity and luminance are the third cluster. They provide the highest contrast and darkest intensity among all groups.

Accounting for the visual characteristics of H&E staining, where pathological features are highlighted by a limited color distribution (i.e., blue/purple and pink), reveals a relationship between the original colors and the resulting grayscale intensity: “Nuclei consistently map to darker pixel intensities, whereas the surrounding tissue structures map to lighter intensities.” This relationship suggests that grayscale conversion does more than just preserve textural information; it also preserves the color stain to a certain degree. The preservation of this diagnostically relevant contrast may be a key reason why the grayscale inputs were able to perform at a level comparable to the RGB baselines on DL, particularly in homogeneous conditions.

### Grayscale conversion algorithm insights

**Fig. 4:**
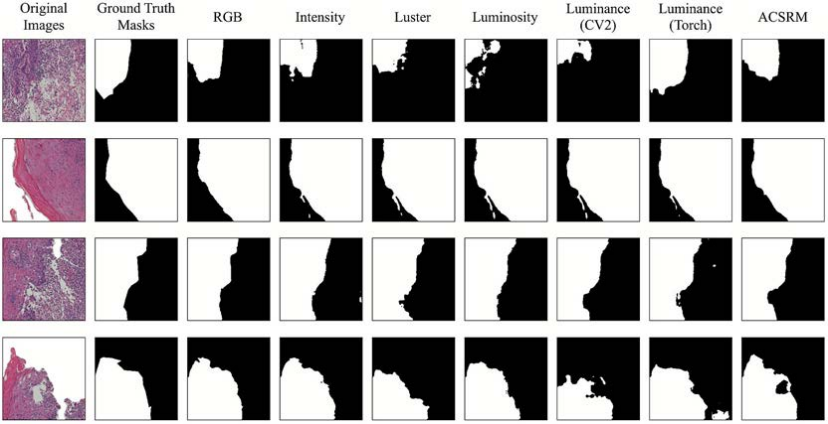
Qualitative visualization (without postprocessing) of UNet using RGB and all grayscale candidates segmentation performance on the OES dataset.

Regarding the single-center scenario, the grayscale candidates largely yielded quantitative performance comparable to their RGB counterparts. To further investigate this parity, we visualized the segmentation outputs of the UNet model (using RGB and all grayscale approaches) on the OES dataset (Fig. 4). Qualitative inspection reveals that ACSRM, in particular, produced segmentation masks that were nearly similar to the RGB baseline, whereas other candidates also demonstrated promising alignment with the ground truth. This analysis validates that even in the absence of significant domain shifts, grayscale conversion effectively preserves the critical morphological cues required for accurate segmentation.

**Fig. 5:**
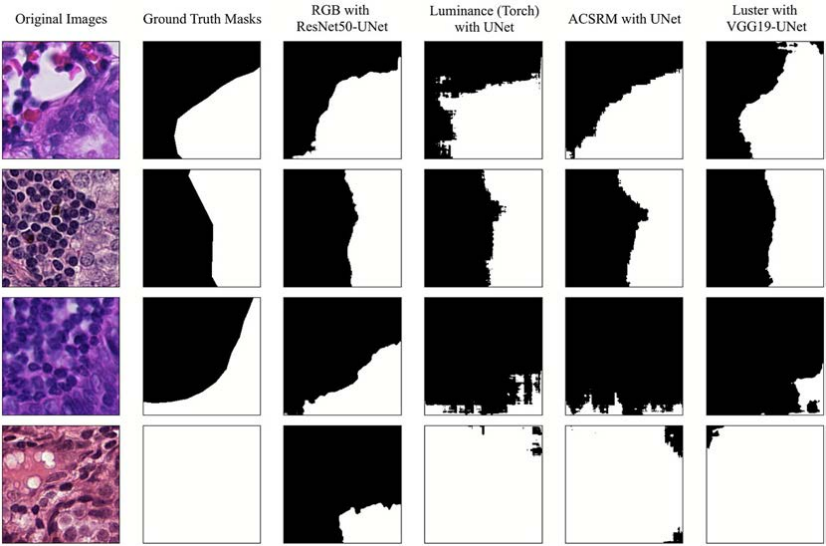
Qualitative visualization (without postprocessing) of top-performing combinations' segmentation performance on The CAMELYON16 dataset.

Furthermore, empirical evidence from the CAMELYON16 results suggests that grayscale formats effectively support DL architectures in extracting generalizable patterns from a multisource H&E image pool. However, to critically examine the specific limitations of RGB versus grayscale inputs, we visualized segmentation examples in Fig. 5. These examples were quantitatively selected to illustrate performance differences. The RGB baseline demonstrated superior segmentation accuracy in patches characterized by highly intensified purple regions. This superiority is likely attributable to the inherent information loss in grayscale conversion when encountering regions with uniform color saturation. In such scenarios, the conversion process resulted in a flattened intensity output, causing a loss of local contrast that hinders the DL's ability to extract distinct textural features, as similarly demonstrated in Fig. 3(f). In contrast, a significant divergence in performance was observed in a region with a lighter and pinkish tone. Whereas the RGB model struggled to generalize across these varying shades, the grayscale champions maintained robust detection, indicating their superior ability to ignore color shifts.

Across all the scenario results, Luster and ACSRM emerged as the most reliable conversion algorithms. Statistical validation via McNemar's test confirmed that these two approaches were the candidates exhibiting a statistically significant difference in predictive behavior compared to their RGB baselines, which also demonstrated superior absolute performance scores under distribution shift scenarios (i.e., cross-scanner and cross-center). Furthermore, they provided promising performance in homogenous settings.

In addition, another technical insight was observed regarding library-specific implementations. Although the CV2 and Torch methods were implemented using the identical standard luminance formula, they yielded different performance outcomes as illustrated in Fig. 4. This discrepancy likely stems from low-level implementation details. This finding highlights the importance of grayscale conversion software libraries in medical AI pipelines to ensure reproducibility.

The primary objective of this study was not to achieve state-of-the-art accuracy, but to evaluate the performance differences between input types. Therefore, all DL models were trained with their default configurations to ensure a fair comparison. Consequently, whereas absolute performance on some tasks—e.g., the Atypia14 dataset—may be suboptimal, the experimental methodology remains valid.

On the Atypia14 dataset, our DL results align with the experiments of Mathew et al.^37^ using the approach of Rezaeilouyeh et al.^38^ The approach utilized shearlet coefficients (i.e., phase and magnitude) and RGB images as features for a general CNN architecture. In contrast, the DL methods achieved high evaluation scores in the experiments requiring a complex pipeline/framework. For example, Xu et al.^39^ employed a CNN-based multiresolution ensemble approach with the plurality voting strategy, and Mathew et al.^37^ proposed a CNN-based framework with a non-relevant nuclei patch filtering and plurality voting strategy.

### Deployment advantages

Beyond improving model robustness, the adoption of grayscale conversion as a standard preprocessing step offers a significant practical advantage for the real-world deployment of DL systems. The primary benefit is a reduction in data dimensionality from a three-channel image (e.g., RGB) to a single-channel image, resulting in approximately one-third of the file size compared to RGB counterparts. This reduction directly leads to lower storage costs and faster data I/O operations. It consequently decreases time-to-upload, which is considered a possible bottleneck in transferring massive WSIs to cloud-based or remote servers where the analysis process takes place. By reducing the data transferred package, a grayscale-based workflow can be faster and more efficient, improving turnaround times.

### Limitations

Whereas this study provides valuable insights into the utilization of grayscale inputs in DL on histopathology tasks, it is important to acknowledge the existing limitations: (1) Our findings are based on limited histopathological tasks. It is crucial to note that these conclusions may not generalize to all tasks, especially for diagnostic challenges that primarily rely on subtle chromatic cues. (2) This study focused on an in-depth systematic exploration of grayscale compared to RGB baselines. A comprehensive analysis would still require benchmarking against established stain normalization techniques. Such a comparison would provide a clearer overview of the most effective strategies for mitigating color variation. (3) The datasets constructed from PANDA and CAMELYON16 were based on a specific sampling of the full WSIs. Thus, the results can vary, if different sampling seeds are utilized or more images are extracted, especially in transformers, which are data-hungry architectures.^32^

## Conclusion

In this study, we systematically investigated the utility of grayscale image conversion for mitigating color variations in DL-based histopathological analysis. Our findings demonstrate that whereas grayscale models perform competitively in homogeneous settings (i.e., single-source data), they exhibited superior generalization in the presence of color variation caused by different scanners and center protocols. In particular, the Luster and our ACSRM algorithms consistently emerged as the most effective techniques, outperforming their RGB baselines in challenging color-distribution-shifted scenarios. By sacrificing color as a sensitive variable, DL models can be guided to learn with better robustness using morphological and structural features, which presents a practical and effective strategy for mitigating domain shift. Combined with inherent deployment advantages (e.g., reduced file size), grayscale conversion represents a promising pathway toward developing more generalizable and deployable AI solutions for clinical digital pathology systems.

To address the constraints imposed by open-source datasets, future research should prioritize the creation of a comprehensive paired-tissue dataset (analogous to Atypia14) that encompasses the full spectrum of cross-source scenarios. Such a resource is essential for conducting rigorous, head-to-head benchmarking between grayscale conversion and well-known stain normalization techniques (e.g., Reinhard,^40^ Macenko,^21^ and generative-based methods^9^^,^^10^). Comparing them directly against grayscale conversion would establish whether the retention of chromatic information provides any tangible benefit for DL generalization compared to the texture-focused representations offered by grayscale algorithms.

## CRediT authorship contribution statement

N. Srisermphoak conceived and designed the study, developed the methodology, performed analysis, drafted and edited the manuscript, and acquired funding. P. Amornphimoltham curated the data and conducted validation experiments. R. Chaisuparat curated data and carried out validation. P. Achararit performed validation. T. Fuangrod performed validation and supervised the work.

## Declaration of large language models in the writing process

During the preparation of this work, the author(s) used Gemni2.5-pro for grammar correction. After using this tool/service, the author(s) reviewed and edited the content as needed and take(s) full responsibility for the content of the publication.

## Declaration of competing interest

The authors declare the following financial interests/personal relationships which may be considered as potential competing interests:

A co-author has been supported by funding from the Chulabhorn Royal Academy under a scholarship contract in the project of Development of Research and Development Talents in Medical Physics and Medical Engineering. Additionally, another co-author has been supported through the Chulabhorn Royal Academy's Fiscal Year 2024 Fundamental Fund of the National Science Research and Innovation Fund (FRB670024/0240, Project Code 198493). If there are other authors, they declare that they have no known competing financial interests or personal relationships that could have appeared to influence the work reported in this article.
